# Supplementary material for: Abnormal calcium release and delayed afterdepolarizations: A comparison of two mathematical models for human ventricular myocytes
Source: PLoS One. 2026 Feb 11;21(2):e0338873. doi: 10.1371/journal.pone.0338873 (PMC12893660; doi:10.1371/journal.pone.0338873)
Supplement: S1 Text — In this document we present the extended methods, results and analysis. (PDF) [file pone.0338873.s012.pdf]

S1 Text: Extended methods and additional analyses

In this document, we provide following details:

- S_{GKr} values required for S_{GCaL}.
- The TP06 and HuVEC15 models with Ca^{2+} overload.
- The Ca^{2+} subsystem and numerical continuation.
- Changes introduced in the TP06 and HuVEC15 myocyte models.
- Subthreshold DADs and I_{Na} inactivation.
- Robustness of our parameter-sensitivity results.
- The effect of the I_{K1} conductance on the DAD amplitude.

S_{GKr} values required for S_{GCaL}

In the main paper, we use a range of values for S_{GCaL}; however, to counter the change in the action potential duration (APD), because of the change in S_{GCaL}, we must adjust the factor S_{GKr}, so we determine the values of S_{GKr} that are required for 4 values of S_{GCaL}, which we use for a straight-line fit (Fig. A), whence we obtain other values of S_{GKr} that should be used for a given value of S_{GCaL}.

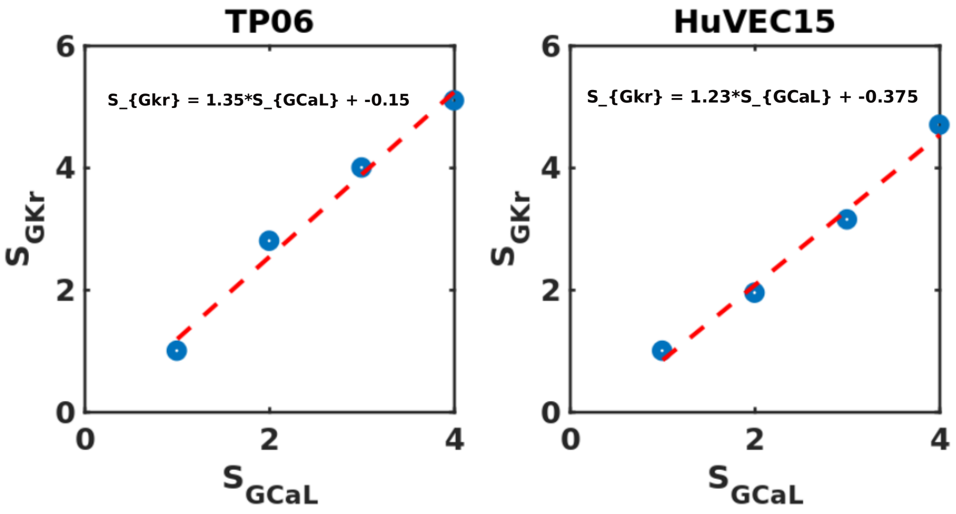

Fig A: Linear fits for the S_{GKr} required for a given S_{GCaL} (see text): for the TP06 (left panel) and HuVEC15 (right panel) models.

The TP06 and HuVEC15 models with Ca^{2+} overload

We increase the calcium load of the TP06 and HuVEC15 models by using $S_{\mathrm{GCaL}} = 2$ (and the corresponding values of S_{GKr} values discussed in the previous Section). We stimulate the myocyte, in both these models, for 500 AP (1 Hz). In this Ca^{2+}-overload condition, the TP06 model triggers an extra systolic calcium spark or late calcium release (LCR) during the AP (see Fig. B(a)), whereas the HuVEC15 model shows a spontaneous calcium release (SCR) in the diastolic interval (Fig.B(b)); these two types of calcium releases force the NCX to the forward mode and, therefore, increase V_{m}. The LCRs leads to V_{m} depolarizations that are similar to EADs, whereas the SCRs lead to DADs.

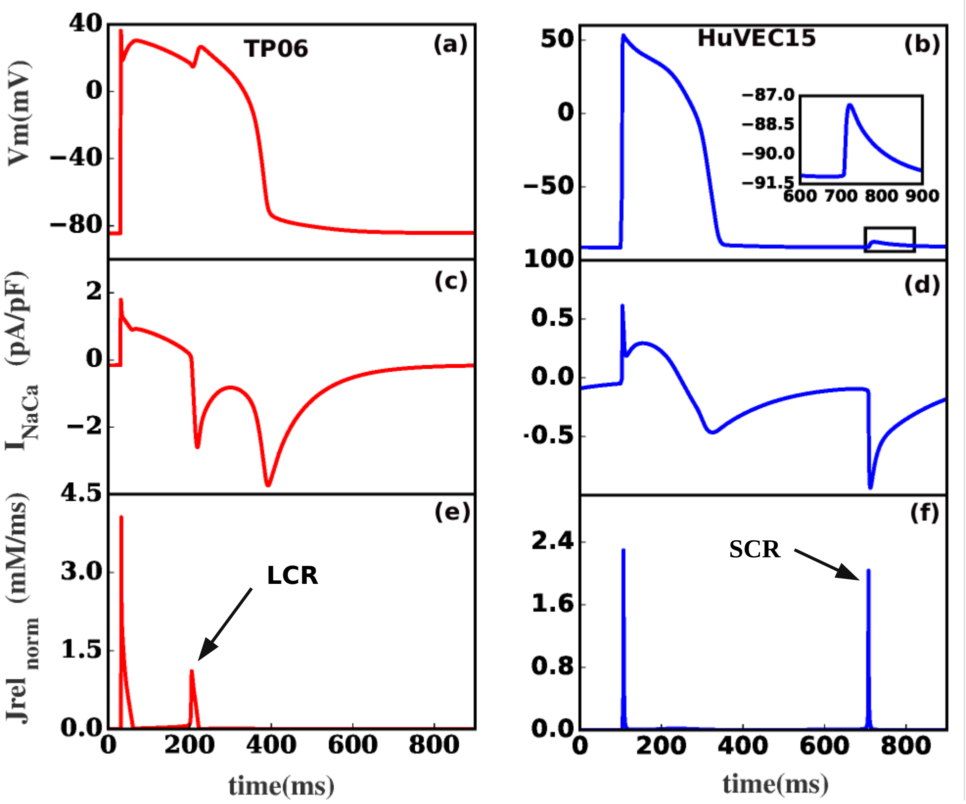

Fig B: Ca^{2+} Sparks and afterdepolarizations, with Ca^{2+} overload, in the TP06 (left) and HuVEC15 (right) models: (a) Ca^{2+} sparks causing EADs in the TP06 model; (b) DADs in the HuVEC15 model; (c) and (d): plots of I_{NaCa} depicting how Ca^{2+} sparks change the direction of NCX from outward to inward; (e) and (f): normalised calcium-release flux from the SR compartment to the subspace (in the TP06 model) or the junctional space (in the HuVEC15 model). We use $S_{\mathrm{GCaL}} = 2$ (and the corresponding values of S_{GKr} values discussed in the previous Subsection).

Thus, we conclude that in the TP06 model, Ca^{2+} overload alone is not sufficient to trigger DADs. Therefore, following the suggestion from Ref. [1], we analyse the Ca^{2+} subsystem of the TP06 model.

The Ca^{2+} subsystem and numerical continuation

Reference [1] proposed that oscillations in the calcium-ion concentration, in the Ca^{2+} subsystem of a cardiomyocyte model, might be associated with DADs. Therefore, we consider the Ca^{2+} subsystems, for the TP06 model, as follows: the Ca^{2+} subsystem contains equations for the Na^{+}-

Ca^{2+} exchanger, the SERCA pump, the RyR release channels, and for the Ca^{2+} concentrations in various compartments. Note that the ODEs for the $V_{\rm m}$ and associated ion-channel dynamics are not part of the Ca^{2+} subsystem; their inclusion complicates the analysis, because we must then consider the full set of ODEs for these models.

The Ca^{2+} subsystem for the TP06 model has 4 ODEs. We provide the detailed ODEs for the Ca^{2+} subsystem for the TP06 model in the Supporting Information.

In our studies of numerical continuation, we follow [2]. The ODEs we consider, e.g., the four ODEs for the TP06 model (see the Supporting Information), are of the form $\dot{u} = F(u, \alpha)$, with $u \in \mathbb{R}^n$ and $\alpha \in \mathbb{R}^m$. An equilibrium u_0 satisfies $F(u_0, \alpha_0) = 0$; and the Jacobian matrix $\mathcal{A} = F_u(u_0, \alpha_0)$ has eigenvalues $\lambda_1, \lambda_2, \ldots, \lambda_N$. A Hopf bifurcation is characterised by the appearance of two, purely imaginary eigenvalues [2]. The Matlab package Matcont [3], which is a numerical-continuation toolbox for ODEs, allows us to obtain the manifold of equilibrium points (we have checked our results by also using the package XPPAUT [4]) and also Hopf-bifurcation points. We illustrate this, for the TP06 model, in Subsection .

$Na_{\rm i}$ overload and the Ca^{2+}-subsystem

The Na^+-Ca^{2+} exchanger (NCX) functions in both the forward and backward directions. During Na^+ overload, it removes 3 Na^+ outside of the cell in exchange for a single Ca^{2+} ion (backward mode); however, an increase in intracellular Ca^{2+} can force the NCX in the opposite direction (forward mode), in which NCX removes 1 Ca^{2+} outside in exchange of 3 Na^+ inside (forward mode). In the reduced model (in the absence of $I_{\rm CaL}$ channels) we use intracellular Na^+ ($Na_{\rm i}$) overload, which forces the Na^+-Ca^{2+} exchanger to the backward mode that overloads the Ca^{2+}-subsystem. The NCX is modeled by the following equation:

$$I_{\rm NaCa} = K_{\rm NaCa} \frac{\exp\left(\frac{\gamma V_{\rm m} F}{RT}\right) {Na_{\rm i}}^3 Ca_{\rm o} - \exp\left(\frac{(\gamma-1) V_{\rm m} F}{RT}\right) {Na_{\rm o}}^3 Ca_{\rm i} \alpha}{({K_{\rm mNai}}^3 + {Na_{\rm o}}^3)(K_{mCa} + Ca_{\rm o})\left(1 + K_{\rm sat} \exp\left(\frac{(\gamma-1) V_{\rm m} F}{RT}\right)\right)} \tag{1}$$

where $V_{\rm m}$ is the membrane potential, $Na_{\rm i}$ the intracellular Na^+ concentration, $Ca_{\rm i}$ the intracellular Ca^{2+} concentration, and

$$\begin{aligned}
\text{constant } \gamma &= 0.35\,; \\
\text{parameter } K_{\rm NaCa} &= 1000.0 \, \frac{pA}{pF}\,; \\
\text{Faraday constant } F &= 96.485 \, \frac{C}{m - mole}\,; \\
\text{Gas constant } R &= 8.314 \, \frac{joule}{mole - K}\,; \\
\text{Temperature } T &= 310 \; K\,; \\
\text{Extracellular calcium concentration } Ca_{\rm o} &= 2 \; mM\,; \\
\text{Extracellular Na concentration } Na_{\rm o} &= 140 \; mM\,; \\
\text{constant } K_{\rm mNai} &= 87.5 \; mM\,; \\
\text{constant } K_{\rm mCa} &= 1.38 \; mM\,; \\
\text{constant } K_{\rm sat} &= 0.1\,.
\end{aligned}$$

For the full description of the Ca^{2+}-subsystem of TP06 model we require, in addition, the following set of equations:

$$
\begin{aligned}
\text{constant } Buf_{\text{c}} &= 0.2\ mM\,; \\
\text{constant } Buf_{\text{sr}} &= 10.0\ mM\,; \\
\text{constant } Buf_{\text{ss}} &= 0.4\ mM\,; \\
\text{constant } EC &= 1.5\ mM\,; \\
\text{constant } K_{\text{buf}_\text{c}} &= 0.001\ mM\,; \\
\text{constant } K_{\text{buf}_\text{sr}} &= 0.3\ mM\,; \\
\text{constant } K_{\text{buf}_\text{ss}} &= 0.00025\ mM\,; \\
\text{constant } K_{\text{up}} &= 0.00025\ mM\,; \\
\text{constant } V_{\text{leak}} &= 0.00036\ s^{-1}\,; \\
\text{constant } V_{\text{rel}} &= 0.102\ s^{-1}\,; \\
\text{constant } V_{\text{sr}} &= 0.001094\ \mu M^3\,; \\
\text{constant } V_{\text{ss}} &= 0.00005468\ \mu M^3\,; \\
\text{constant } V_{\text{xfer}} &= 0.0038\ ms^{-1}\,; \\
\text{constant } V_{\text{maxup}} &= 0.006375 \frac{mM}{ms}\,; \\
\text{constant } k_1' &= 0.15\ (M^2 - ms)^{-1}\,; \\
\text{constant } k_2' &= 0.045\ (mM - ms)^{-1}\,; \\
\text{constant } k_3 &= 0.06\,; \\
\text{constant } k_4 &= 0.005\,; \\
\text{constant } max_{\text{sr}} &= 2.\,;
\end{aligned}
$$

$$
\begin{aligned}
\text{constant } min_{\text{sr}} &= 1.0\,; \\
\text{constant } C_{\text{m}} &= 0.185\ \mu F\,; \\
\text{constant } V &= -85.23\ mV\,; \\
\text{constant } V_{\text{c}} &= 0.016404\ \mu m^3\,; \\
\text{constant } \alpha &= 2.5\,;
\end{aligned}
$$

$$I_{\text{leak}} = \begin{cases} V_{\text{leak}}\left(Ca_{\text{SR}} - Ca_{\text{i}}\right), & \text{if isolated myocyte}\,; \\ 0, & \text{tissue}\,; \end{cases} \tag{2}$$

$$I_{\text{up}} = \frac{V_{\text{maxup}}}{1 + \left(\frac{K_{\text{up}}}{Ca_{\text{i}}}\right)^2}\,; \tag{3}$$

$$I_{\text{rel}} = \left(V_{\text{rel}}.O + V_{\text{RyRL}}\right)\left(Ca_{\text{SS}} - Ca_{\text{SS}}\right)\,; \tag{4}$$

$$V_{\text{RyRL}} = 0.00018\,;$$

$$I_{\text{xfer}} = V_{\text{xfer}}\left(Ca_{\text{SS}} - Ca_{\text{i}}\right)\,; \tag{5}$$

$$O = \frac{k_1 {Ca_{\text{SS}}}^2 \bar{R}}{k_3 + k_1 {Ca_{\text{SS}}}^2} \tag{6}$$

$$\frac{d\bar{R}}{dt} = -k_2 Ca_{\text{SS}}\bar{R} + k_4\left(1 - \bar{R}\right)\,; \tag{7}$$

$$Ca_{\text{i}_{\text{bufc}}} = \frac{1.0}{1.0 + \dfrac{Buf_c.K_{\text{buf}_\text{c}}}{\left(Ca_{\text{i}} + K_{\text{buf}_\text{c}}\right)^2}}\,; \tag{8}$$

$$Ca_{\text{sr}_{\text{bufsr}}} = \frac{1.0}{1.0 + \dfrac{Buf_{sr}K_{\text{buf}_\text{sr}}}{\left(Ca_{\text{SR}} + K_{\text{buf}_\text{sr}}\right)^2}}\,; \tag{9}$$

$$Ca_{\text{ss}_{\text{bufss}}} = \frac{1.0}{1.0 + \dfrac{Buf_{\text{ss}}K_{\text{buf}_\text{ss}}}{\left(Ca_{\text{SS}} + K_{\text{buf}_\text{ss}}\right)^2}}\,; \tag{10}$$

$$\frac{dCa_{\text{i}}}{dt} = Ca_{\text{i}_{\text{bufc}}}\left(\left(I_{\text{leak}} - I_{\text{up}}\right)\frac{V_{\text{sr}}}{V_{\text{c}}} + I_{\text{xfer}} - 2.I_{\text{NaCa}}.C_{\text{m}}/\left(2V_{\text{c}}F\right)\right)\,; \tag{11}$$

$$\frac{dCa_{\text{SR}}}{dt} = Ca_{\text{sr}_{\text{bufsr}}}\left(I_{\text{up}} - \left(I_{\text{rel}} + I_{\text{leak}}\right)\right)\,; \tag{12}$$

$$\frac{dCa_{\text{SS}}}{dt} = Ca_{\text{ss}_{\text{bufss}}}\left(I_{\text{rel}}\frac{V_{\text{sr}}}{V_{\text{ss}}} - I_{\text{xfer}}\frac{V_{\text{c}}}{V_{\text{ss}}}\right)\,; \tag{13}$$

$$k_1 = \frac{k_1'}{k_{\text{casr}}}\,; \tag{14}$$

$$k_2 = k_2' k_{\text{casr}}\,; \tag{15}$$

$$k_{\text{casr}} = max_{\text{sr}} - \frac{max_{\text{sr}} - min_{\text{sr}}}{1 + \left(\dfrac{EC}{Ca_{\text{SR}}}\right)^2}\,. \tag{16}$$

Nonlinear analysis of the Ca^{2+}-subsystem

In Fig. C(a), we illustrate that, under the control parameter $Na_{\text{i}} = 65$ mM, the typical value attained by Ca_{SR} is 17 mM. Despite this notable Ca^{2+}-overload, there were no indications of Ca^{2+}-oscillations. To search for the parameters for Ca^{2+}-oscillations we begin with this steady-state value and perform equilibrium continuation, by using the Matlab package Matcont [3], to obtain the dependence of this equilibrium value on Na_{i}, which we show via the purple full curve in Fig. C(b). We also find that a pair of neutral-saddle equilibrium points appear beyond a threshold value of Na_{i} that we indicate by a blue point in Fig. C(b), dashed part of the curve shows the dependence of the neutral-saddles on Na_{i}; this curve meets the purple equilibrium at

the red Hopf critical point. Beyond this critical value of Na_i, there are no critical points and the long-time behavior of Ca_SR is oscillatory, because of a limit cycle that results from an Andropov-Hopf bifurcation. In Fig. C(c) we present an illustrative plot of the temporal oscillations in Ca_SR at $Na_\mathrm{i} = 70$ mM. Oscillations also occur in the total calcium content (free and buffered) Ca_Tot and Ca_SS as we show in Figs. C(d) and (e), respectively, for the representative value $Na_\mathrm{i} = 70$ mM; the underlying limit cycle's projection is shown in Fig. C(f) via the red, closed curve in the Ca_SR - Ca_SS plane. Thus, we have shown that the TP06 Ca^{2+}-subsystem can show calcium oscillations, which lead, in turn, to DADs in the myocyte AP.

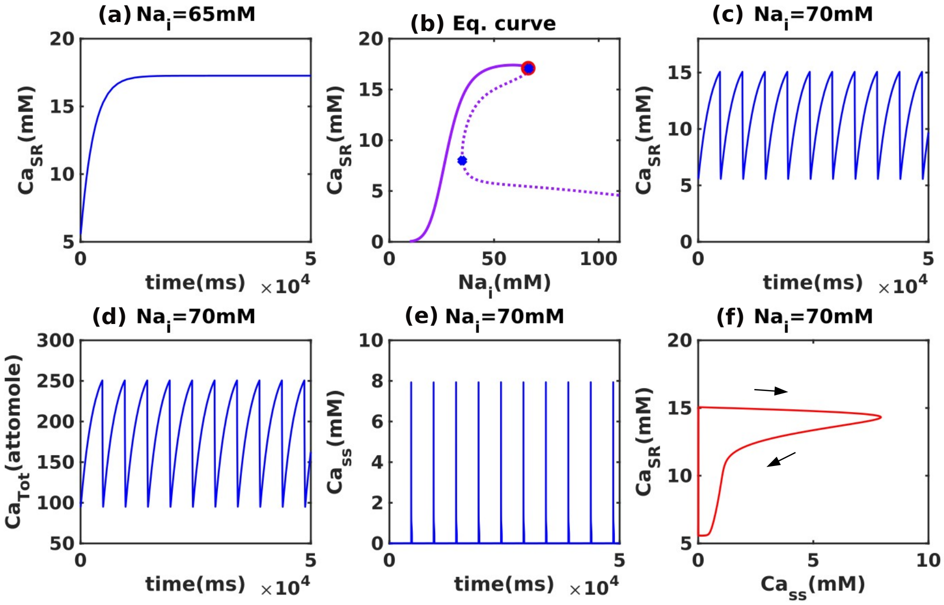

Fig C: Equilibrium continuation and Ca^{2+} oscillations: (a) Plot of Ca_SR versus time t, at $Na_\mathrm{i} = 65$ mM, showing saturation at long time to an equilibrium concentration; by using equilibrium continuation (see text) we detect an Andropov-Hopf bifurcation in the TP06 Ca^{2+}-subsystem. (b): equilibrium curve exhibiting the dependence of fixed points on the parameter Na_i. The solid line represents the stable equilibrium values. The red dot at $Na_\mathrm{i} = 66.48$ mM shows the transition of the system to an oscillatory state, and the blue dots at the turning points show the emergence and vanishing of two extra equilibrium points. (c): Ca_SR oscillations at $Na_\mathrm{i} = 70$ mM; (d): total calcium content (free and buffered) Ca_Tot oscillations at $Na_\mathrm{i} = 70$ mM; (e): Ca_SS oscillations at $Na_\mathrm{i} = 70$ mM; (f): Ca_SR and Ca_SS oscillations.

Hence, our inference from this subsection is that achieving Ca^{2+}-oscillations in TP06 Ca^{2+}-subsystem (and DADs in full TP06 model) necessitates an exceedingly high (and unrealistic) Ca^{2+}-overload. To induce DADs under realistic parameters, we introduced a constant V_RyRL that simulates Ca^{2+} leak from SR to cytosol through RyR (see Eq. 5 in main text).

Role of the RyR leak current in the TP06 and HuVEC15 models

In both these models, the opening of the RyRs is modeled via the Ca-induced-Ca release (CICR). The calcium availability inside the SR stores (Ca_SR and Ca_SRrl for TP06 and HuVEC15 models, respectively) and the subspaces outside the RyR, also called triggers (Ca_SS and Ca_jnc for TP06 and HuVEC15 models, respectively) both modulate the opening probability of the RyR. If the trigger is not enough during the diastole, then Ca^{2+} sparks and DADs do not occur. Therefore,

a small calcium leak, through the closed RyRs, suffices for $[Ca^{2+}]_{\rm SS}$ to reach the threshold trigger levels required for SCRs and DADs. Equation 5 (see main text) describes such leak for TP06 model. This kind of RyR leak is already present in the HuVEC15 model as follows:

$$
\begin{aligned}
I_{\rm rel} &= \frac{J_{\rm rel}}{V_{\rm SRrl}}\,; V_{\rm SRrl} = \; 300 \text{ fL}\,; \\
V_{\rm rel} &= \frac{P_{\rm RyR}}{V_{\rm SRrl}}\,; P_{\rm RyR} = \; 5191 \text{ fL/mS}\,; \\
J_{\rm rel} &= P_{\rm RyR}(p_{\rm ORyR} + 0.000075)([Ca^{2+}]_{\rm SRrl} - [Ca^{2+}]_{\rm jnc})\,;
\end{aligned}
\tag{17}
$$

$J_{\rm rel}$ is the CICR current , $P_{\rm RyR}$ the rate constant, factor $P_{\rm RyR} \times 0.000075$ is the RyR leak current, $p_{\rm ORyR}$ is the RyR opening probability, $[Ca^{2+}]_{\rm SRrl}$ and $[Ca^{2+}]_{\rm jnc}$ are, respectively, the molar calcium concentrations in the junctional space and release compartments of the SR. The RyR leak plays a crucial role in triggering SCRs and DADs, in both TP06 and HuVEC15 models.

We re-performed the continuation analysis on the TP06 model with the leaky RyR and compared the results to a control case where these changes were not implemented. In Figs. D (a) and (b) we show, for the TP06 Ca^{2+}-subsystem, how the equilibrium-continuation curves and Hopf point, shown in Fig. C(b), change as we tune the scale factor $S_{\rm Vmaxup}$ (for $V_{\rm maxup}$), both without and with the RyR leak, respectively. In particular, the Hopf point moves as we change the scale factor $S_{\rm Vmaxup}$; the Hopf points are shown in blue, red, cyan, black, and purple for $S_{\rm Vmaxup} = 1.4, 1.2, 1.0, 0.8,$ and 0.6, respectively.

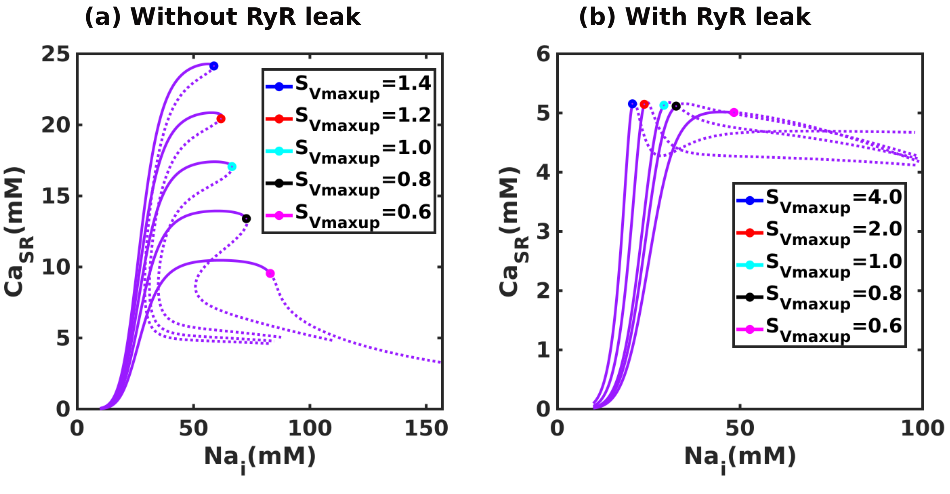

Fig D: Andronov-Hopf bifurcations and their SERCA dependence: To detect the change in the Hopf-bifurcation point with other parameters, such as the SERCA-pump uptake rate $S_{\rm Vmaxup}$, we perform equilibrium continuation for different values of $S_{\rm Vmaxup}$. (a): $Ca_{\rm SR}$ versus $Na_{\rm i}$ plots of equilibrium-continuation curves in purple and Hopf points on them; (b): $Ca_{\rm SR}$ versus $Na_{\rm i}$ plots of equilibrium-continuation curves and Hopf points on them for various values of $S_{\rm Vmaxup}$, in the presence of the RyR leak. In both the plots, an increase in $S_{\rm Vmaxup}$ shifts the Hopf point towards large values of $Ca_{\rm SR}$ and low values of $Na_{\rm i}$; by comparing (a) and (b), we can infer how the RyR leak affects the $Ca_{\rm SR}$ and $Na_{\rm i}$ requirements for this Andronov-Hopf bifurcation.

By comparing Figs. D (a) and (b), we see that the RyR leak lowers the values of $Ca_{\rm SR}$ and $Na_{\rm i}$ at which the Hopf-point occurs. Moreover, including thIS RyR leak in the full TP06 model

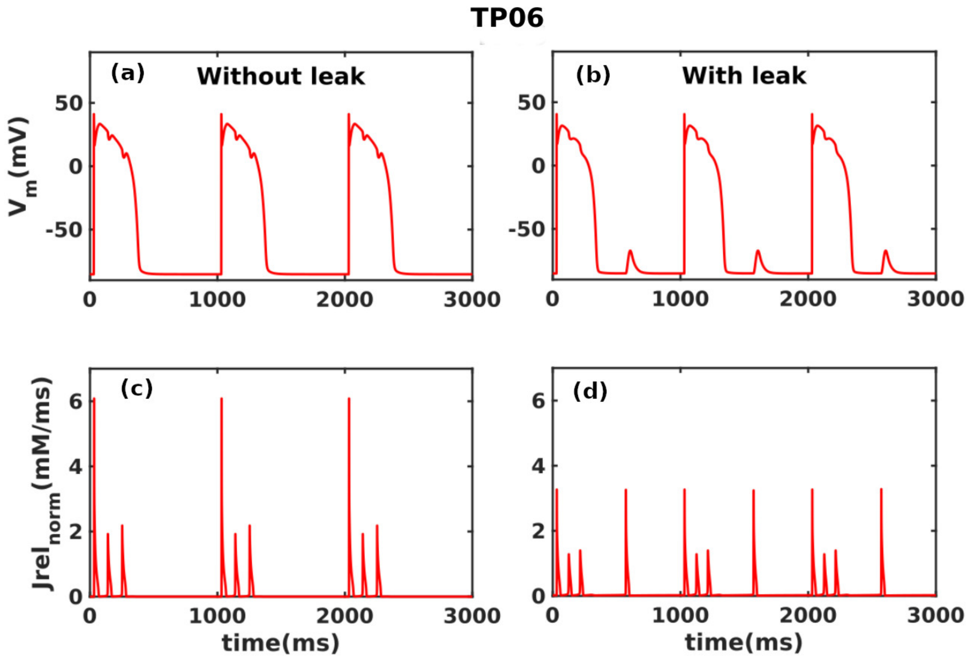

Fig E: The role of the RyR leak in triggering DADs in the TP06 model: (a): No DADs are observed without the RyR leak; (b) after modifying the equations to introduce an RyR leak this model triggers DADs.

indeed leads to a DAD response (see Fig. E).

Distribution of NCX channels in HuVEC15 model

Without any additional changes, we observe the DADs in the HuVEC15 model but the amplitude of the DADs are upto 2 mV. To increase the amplitude of the DADs in HuVEC15, we introduce the following change; while preserving the overall density of NCX in the various compartments of the model, we increase the fraction of NCX in the intermediate zone (iz) of the HuVEC15 model (f_{NCX}).

Cardiac myocytes have multiple compartments with different calcium concentrations; this is usually called calcium compartmentalization [5]. The distribution of ion channels in each compartment is different. The HuVEC15 model has 90% of ion channels in the bulk cytosol region, and the remaining 10% are in the intermediate zone (iz near RyRs in Fig. 1 in the main paper). However, the fraction of NCX ion channels near the RyRs may be up to 45% Ref. [6]. Therefore, in the HuVEC15 model, we increase the fraction of NCX channels in the iz to 25% from 10%. This modification in the NCX distribution increases the calcium-to-voltage coupling gain [7] of the myocyte and, thereby, increases the DAD amplitude for a given SCR.

Subthreshold DADs and I_{Na} inactivation

We demonstrate the role of subthreshold and multi-blip DADs in the inactivation of the Na^+ channel. The study of Ref. [8] has suggested that subthreshold DADs can inactivate the Na^+ channel and can act as a substrate for promoting conduction block. In particular, we compare the effects of multi-blip and subthreshold DADs on the inactivation gates of the I_{Na}. In Fig. F(a) we show that the subthreshold DADs can inactivate the I_{Na} gates, i.e., h (fast-inactivation) and

j (slow-inactivation) gates in the TP06 model, as shown by the plots of the product $h * j$ of these gates (Fig. F(c)). Similarly, multi-blip DADs (Fig. F(b)) inactivate fast I_{Na} on multiple occasions (see Fig. F(d)).

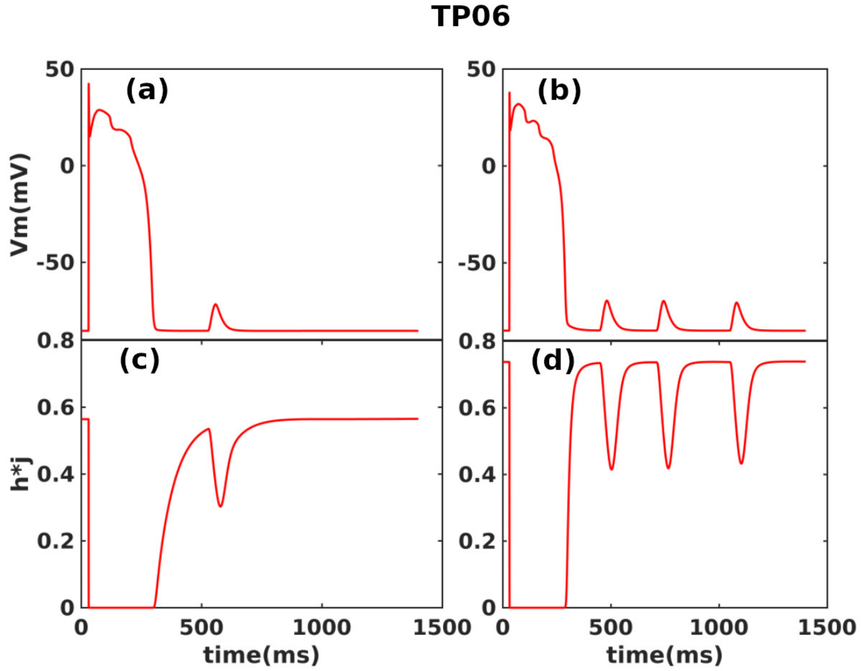

Fig F: Comparison of subthreshold and multi-blip DADs based on I_{Na} channel inactivation:. The top panels show TP06-model membrane potentials for subthreshold DADs (left) and multi-blip DADs (right). The bottom panels show the product of I_{Na} inactivation gates [$h * j$ (see text)] for these DADs: (a) Subthreshold DADs; (b) multi-blip DADs; the product of inactivation gates ($h * j$) for (c) subthreshold DADs and (d) multi-blip DADs.

Robustness of our parameter-sensitivity results

We demonstrate that our parameter-sensitivity analyses are robust for a range of pacing frequencies in the sense that they yield results similar to those in the main paper, even if we use a different pacing frequency for stimulating the myocyte [see Figs. G and H].

Role of I_{K1} conductance in DAD amplitude

In the main paper, we have discussed the three types of DADs that can occur in the TP06 and HuVEC15 models and the parameters that control the incidence frequencies and amplitudes of these DADs. We know that NCX increases V_{m} in response to CICR; the ratio of the rise in V_{m}, in response to the SCR (or Ca_{i}) amplitude, is known as calcium-voltage coupling gain [7]. In the context of DADs, given the amplitudes of SCRs, the calcium-voltage coupling gain, during the diastolic interval, primarily depends on two currents, namely, I_{NaCa} and I_{K1}; the former competes against the latter. Therefore, the interplay of the parameters S_{KNaCa} and S_{GK1} controls the maximum amplitude that a DAD reaches. A loss-of-function of the I_{K1} channel has been identified in some conditions such as Anderson's syndrome [9]; therefore, we use a reduced value of G_{K1} to simulate this effect on the DAD amplitude. In Fig. I we show that a reduction in G_{K1} can amplify subthreshold DADs so that they become suprathreshold DADs.

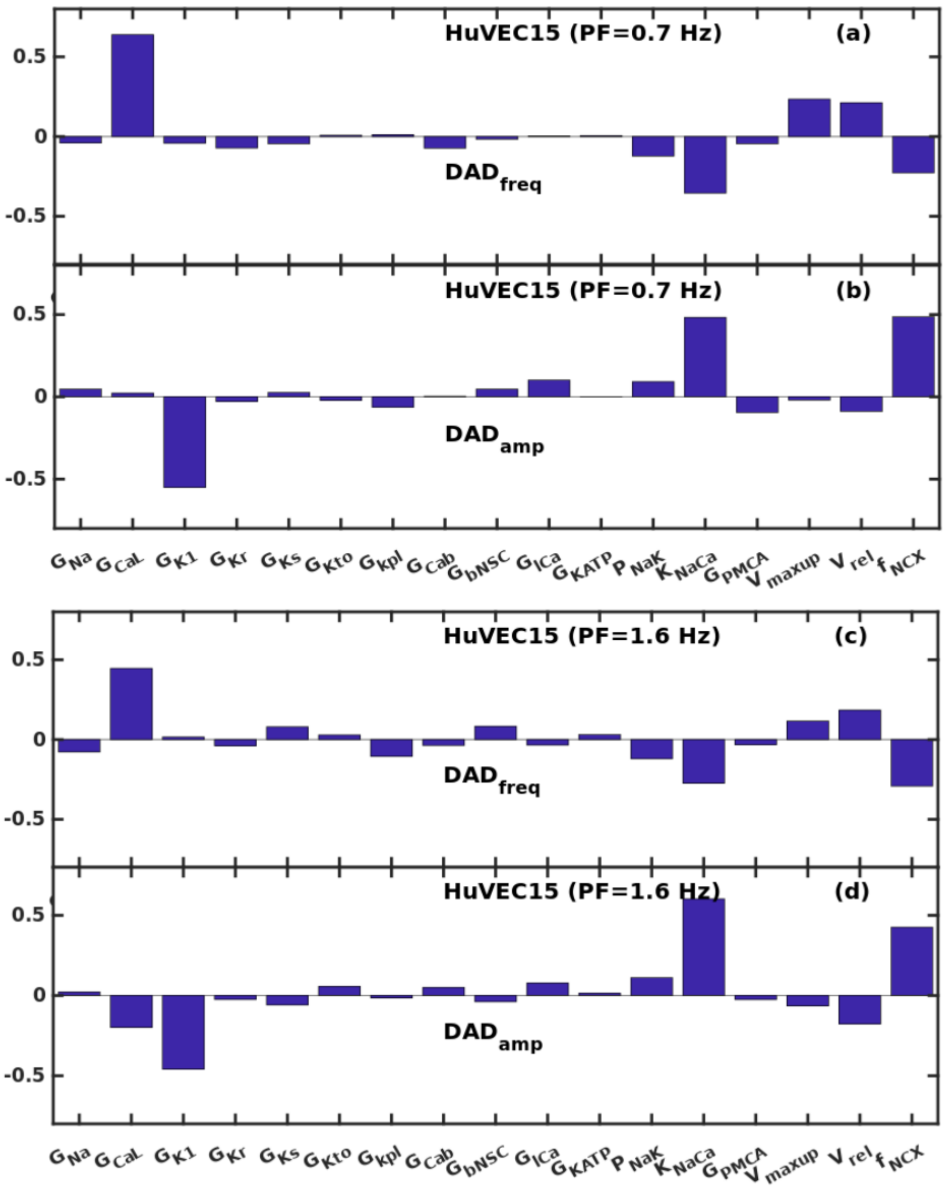

Fig G: Parameter sensitivity at 0.7 Hz and 1.6 Hz pacing frequencies (HuVEC15 model). (a) DAD-frequency sensitivity plot at 0.7 Hz; (b) DAD-amplitude sensitivity plot at 0.7 Hz; (c) DAD-frequency sensitivity plot at 1.6 Hz; (b) DAD amplitude sensitivity plot at 1.6 Hz.

We also know that V_{maxup} can reduce the coupling interval between DADs and the previous AP. Therefore, a reduction in G_{K1} can lead to a sustained incidence of triggered activity if this reduction is combined with multi-blip DADs as the coupling interval between DADs and the AP is very small in the case of multi-blip DADs.

References

[1] Fink M, Noble PJ, Noble D. Ca2+-induced delayed afterdepolarizations are triggered by dyadic subspace Ca2+ affirming that increasing SERCA reduces aftercontractions. American Journal of Physiology-Heart and Circulatory Physiology. 2011;301(3):H921-35.

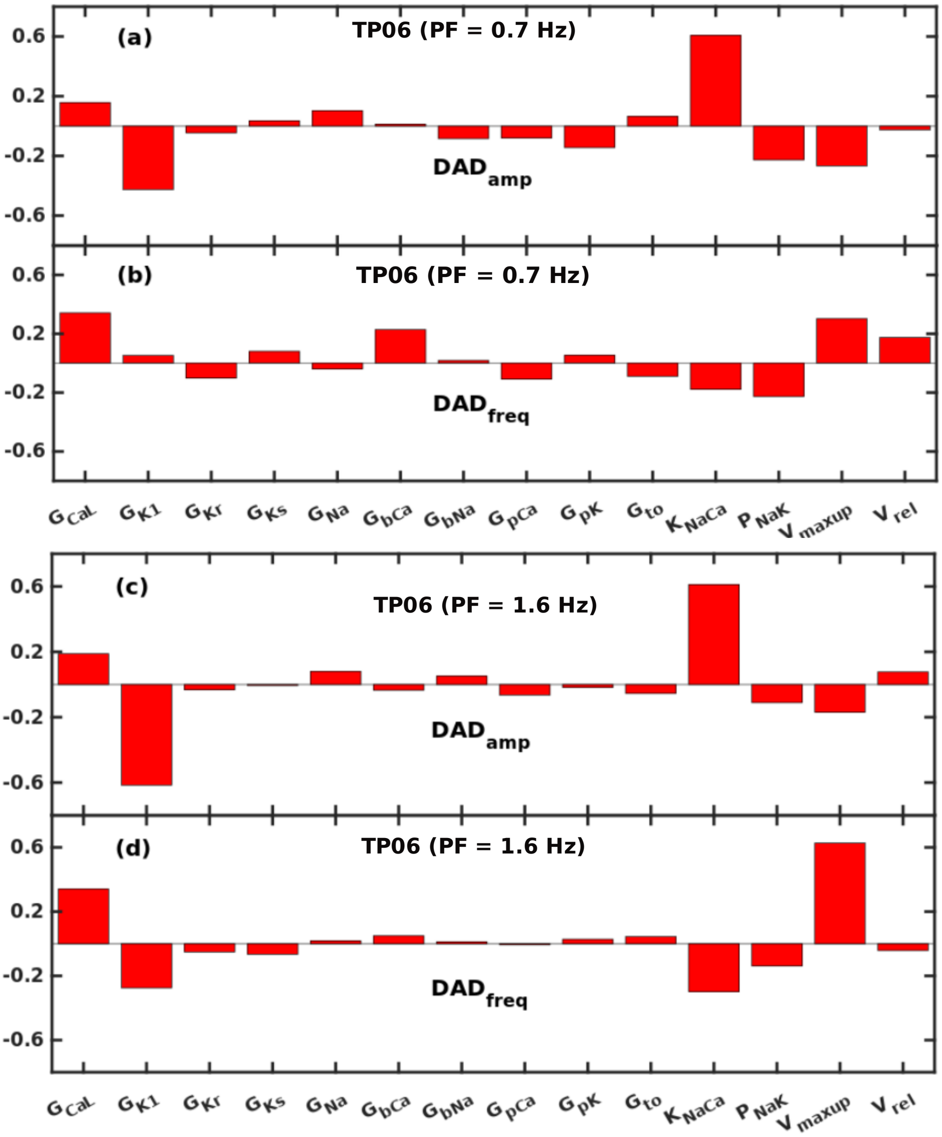

Fig H: Parameter sensitivity at 0.7 Hz and 1.6 Hz pacing frequencies (TP06 model). (a) DAD-frequency sensitivity plot at 0.7 Hz; (b) DAD-amplitude sensitivity plot at 0.7 Hz; (c) DAD-frequency sensitivity plot at 1.6 Hz; (b) DAD amplitude sensitivity plot at 1.6 Hz.

[2] Kuznetsov YA. Codim 1 bifurcations of n-dimensional ODEs; 2019. Available from: `https://webspace.science.uu.nl/~kouzn101/USS2.pdf`.

[3] Dhooge A, Govaerts W, Kuznetsov YA. MATCONT: a MATLAB package for numerical bifurcation analysis of ODEs. ACM Transactions on Mathematical Software (TOMS). 2003;29(2):141-64.

[4] Ermentrout B. XPPAUT 5.0-the differential equations tool. University of Pittsburgh, Pittsburgh. 2001.

[5] Langer GA. Myocardial calcium compartmentation. Trends in Cardiovascular Medicine. 1994;4(3):103-9.

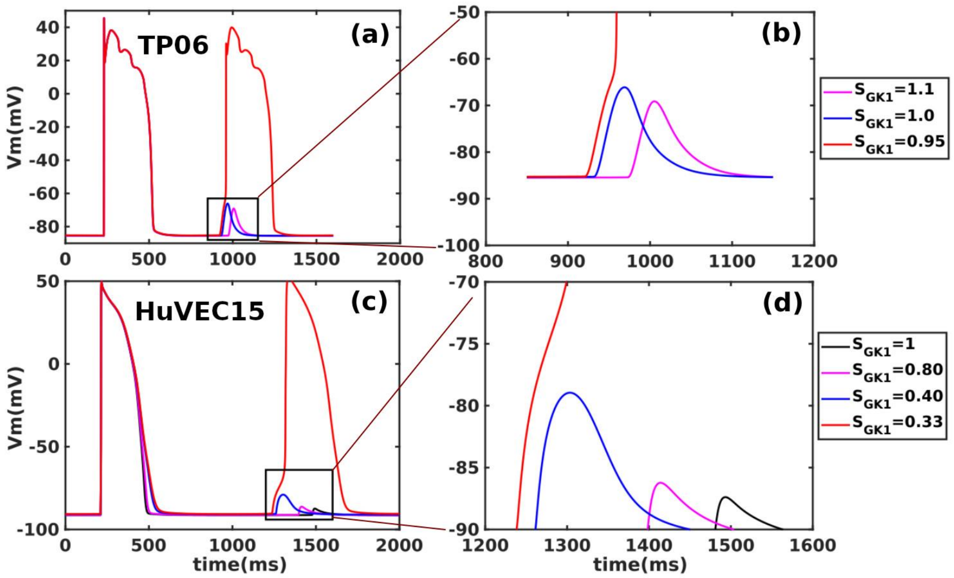

Fig I: Effect of G_{K1} on the DAD$_{\mathbf{amp}}$ (see text): (a) for the TP06 model; (b) expanded version of the inset in (a); (c) for the HuVEC15 model; (d) expanded version of the inset in (c). In both these models a reduction in G_{K1} converts subthreshold DADs to suprathreshold amplitude.

[6] Chu L, Greenstein JL, Winslow RL. Modeling Na+-Ca2+ exchange in the heart: Allosteric activation, spatial localization, sparks and excitation-contraction coupling. Journal of molecular and cellular cardiology. 2016;99:174-87.

[7] Maruyama M, Joung B, Tang L, Shinohara T, On YK, Han S, et al. Diastolic intracellular calcium-membrane voltage coupling gain and postshock arrhythmias: role of purkinje fibers and triggered activity. Circulation research. 2010;106(2):399-408.

[8] Liu MB, de Lange E, Garfinkel A, Weiss JN, Qu Z. Delayed afterdepolarizations generate both triggers and a vulnerable substrate promoting reentry in cardiac tissue. Heart rhythm. 2015;12(10):2115-24.

[9] Verkerk AO, Veldkamp MW, Baartscheer A, Schumacher CA, Klöpping C, van Ginneken AC, et al. Ionic mechanism of delayed afterdepolarizations in ventricular cells isolated from human end-stage failing hearts. Circulation. 2001;104(22):2728-33.
