# Supplementary material for: Abnormal calcium release and delayed afterdepolarizations: A comparison of two mathematical models for human ventricular myocytes
Source: PLoS One. 2026 Feb 11;21(2):e0338873. doi: 10.1371/journal.pone.0338873 (PMC12893660; doi:10.1371/journal.pone.0338873)
Supplement: Attachment — Submitted filename: Review_responses_10-12-2024.pdf [file pone.0338873.s013.pdf]

AR: Author’s response

1. Please ensure that your manuscript meets PLOS ONE's style requirements, including those for file naming. The PLOS ONE style templates can be found at https://journals.plos.org/ https://journals.plos.org/

2. Please note that PLOS ONE has specific guidelines on **code sharing** for submissions in which author-generated code underpins the findings in the manuscript. In these cases, all author-generated code must be made available without restrictions upon publication of the work. Please review our guidelines at https://journals.plos.org/ and ensure that your code is shared in a way that follows best practice and facilitates reproducibility and reuse.

3. Please note that funding information should not appear in any section or other areas of your manuscript. We will only publish funding information present in the Funding Statement section of the online submission form. Please **remove any funding-related text** from the manuscript.

4. In the online submission form, you indicated that “All the data will be made avaialbe upon request”. All PLOS journals now require all data underlying the findings described in their manuscript to be freely available to other researchers, either 1. In a public repository, 2. Within the manuscript itself, or 3. **Uploaded as supplementary information**. This policy applies to all data except where public deposition would breach compliance with the protocol approved by your research ethics board. If your data cannot be made publicly available for ethical or legal reasons (e.g., public availability would compromise patient privacy), please explain your reasons on resubmission and your exemption request will be escalated for approval.

5. Please upload a copy of **Supporting Information** which you refer to in your text on **page 14**.

Additional Editor Comments:
All Reviewers commented on the fact that the **aims and rationale of the manuscript need to be better defined**, and that there needs to be an **improved positioning** of the manuscript concerning published literature regarding model analysis of DADs. All comments of the Reviewers should be addressed in a revised version of the manuscript.

Please use the space provided to explain your answers to the questions above. You may also include additional comments for the author, including concerns about dual publication, research ethics, or publication ethics. (Please upload your review as an attachment if it exceeds 20,000 characters)

Reviewer #1

The paper, "Abnormal Calcium Release and Afterdepolarizations: A Comparison of Two Mathematical Models for Human Ventricular Myocytes", investigates delayed

afterdepolarizations (DADs) in cardiac myocytes, which are linked to fatal arrhythmias. The study compares two human ventricular myocyte models—the ten Tusscher-Panfilov TP06 model and the HuVEC15 model. The authors analyze the effects of various electrophysiological parameters on DADs using mathematical modeling. Through continuation analysis, they reveal how calcium concentration changes in the sarcoplasmic reticulum (SR) and calcium pumps affect DADs. The study identifies three types of DADs —subthreshold, suprathreshold, and multi-blip—and uses sensitivity analysis to determine which parameters significantly influence these DAD types. Simulations demonstrate how patches of DAD cells in tissue can trigger premature ventricular complexes (PVCs), a key marker for arrhythmias. The paper emphasizes the role of the Na+/Ca2+ exchanger in suppressing DADs in the TP06 model, offering insights into potential therapeutic targets for preventing arrhythmia.

AR: We thank the reviewer for this assessment of our work.

Major concerns:

1) It was not clear to me what new question this study addressed. There is an extensive body of work on model analysis of DAD's what new question is addressed here. Please better motivate why two models were analysed and why you chose these two models.

In two human ventricular myocyte models, we present DADs of three types, and characterise them, based on few key features, such as the amplitude and frequency of the DADs, for which we use sensitivity analysis to highlight the parameters that help us to identify these features.

We study these common-pool models (TP06 and HuVEC15) as they prove to be apt for tissue simulations, including the pathophysiological conditions with evolving parameters, such as acidosis, during which these parameters evolve as the pH value of the tissue changes (line numbers 53-58 in our manuscript).

We compare two mathematical models to highlight the crucial features, which are common and different in these models, as far as their abilities to produce DADs is concerned. From the possible models, we selected the TP06 and HuVEC15 models, because the TP06 model, a well-established model, has been extensively validated for tissue simulations. We chose the HuVEC15 model as it was one of the recent models that is capable of reproducing Delayed Afterdepolarizations (DADs).

We have updated the manuscript by adding lines 45-51.

2) Were differences explainable by underlying data or model structure?

The two crucial differences between these mathematica models are as follows:

(i) When we scale up G_CaL, keeping the APD intact (by increasing G_Kr), this increases the inward Ca current during the plateau phase of I_CaL; this leads to Late Calcium Releases (LCRs) in the TP06 model; by contrast, there are no such LCRs in the HuVEC15

model. We had already written this in our paper. We had also note in the paper (lines 339-340 and 411-415) that:

“Under Ca2+ overload, the TP06 model shows LCRs, which lead to EAD-type depolarizations without reopening the I_CaL channel; such LCRs have been reported in Refs. [53], [54], [55], [56], [57]; however, the HuVEC15 model does not exhibit these LCRs.”

These LCRs arisee from the fast recovery of RyRs from inactivation, and SR replenishment because of a high uptake rate of the SERCa pump.

(ii) A small increase in NCX was helpful in enhancing the amplitude of DADs in the TP06 model; however, too large an increase in the NCX in the TP06 model led to the termination of the DADs. By contrast, no such behavior was present in the HuVEC15 model.

As pointed out in our manuscript this crucial difference between these models could be because of LCRs.

3) There did not appear to be any validation of the models. I do not believe the TT model (not sure about HuVEC15) were designed to study DAD’s can you confirm through comparison to experimental data that they represent the key mechanism?

The TT model (in our terminology TP06 model) was not developed for DADs; however, it contains necessary features [see Ref. 37, by Fink *et al.* in the Manuscript] to produce spontaneous Ca release and DADs, therefore, this model has been used in previous computational studies with DADs [see Refs. 17 and 18 in the manuscript].

The TP06 model hass the following key properties that can lead to DADs:

(1): Spontaneous Ca release: Using Ca overload and few changes in parameters, the TP06 model shows diastolic Ca releases which are not initiated by I_CaL.

(2): The necessary physiological components for DADs: The role of physiological factors such as V_maxup, I_NaCa and I_K1, all are present in the model; these factors are crucial for the the initiation, upstroke and recovery phase of DADs.

Query:

1. Does the Hinch model include calcium induced inactivation of the L-type Ca channel. Does this impact the generation of DADs.

AR: Yes. The Hinch model and the HuVEC15 model, the latter considered in our study, do take into account the calcium induced inactivation of the L-type Ca channel. If not inactivated these (LCC) can reactivate the RyR during the plateau phase of the APs, therefore depleting the SR stores, and reducing the chances of DADs during the diastole.

2. The mesh is anisotropic with 250 um edge length in plane and 1000 um edge length in the z-direction. This seems very large. If the waves are flat, it may not have an impact but may be good to check.

AR: In our study, the grid-size we have taken is 0.025 cm, which is same in all the three directions.

3. The TT models are known to have a high APD depedence on I_Ks, as opposed to I_Kr, did this mean you needed much larger changes in TT I_Kr to maintain APD duration than HuVEC15. Could this impact your results?

AR: The TT (TP06) model has a higher APD depedence on I_Ks, than on I_Kr. As presented in Figs. 2 and 15. In both TP06 and HuVEC15 models, for consistency we use I_Kr to maintain the APD; and the I_Kr required to control the APD is higher in HuVEC15 than in the TP06 model; however, this does not have a significant impact on our results; this is reflected in the sensitivity dependence [see Fig. 4] for both the models.

4. The tissue models have each vertex representing large numbers of cells. Is this a reasonable model for DADs which are expected to be random, but in the current model you are assuming a large number of cells are synchronised, what is the mechanisms for this??

AR: As this is a continuum model, the DAD region is represented as a clump of DAD myocytes, where each grid point is more than an individual myocyte. The synchronization of the DAD myocytes in the patch is due to the pacing from the previous plane wave (see Ref. 18 in the manuscript). Such continuum models are useful in the studies with interactions of a plane wave (due to normal excitations) and a target wave (due to arrythmogenic origin such as DADs). We have stated this in line nos. 354-355, in the updated version of the manuscript.

Minor:

"electrogenic exchange of cytosolic Ca2+ against the extracellular Na+" → Change "against the" to "for three".

We have corrected this and included it in the updated version of the manuscript.

"The NCX and SERCA work together for the regulation of cytosolic Ca2+" → Consider revising "for the regulation" to "to regulate".

We have included it in the updated version of the manuscript.

"calcium-overload conditions and shown the following" → Add "have" before "shown".

We have included this in the updated version of the manuscript.

****************************** **Reviewer #2** ***

Here, Drs. Roshan and Pandit compare the determinants of DAD due to spontaneous calcium release events from the sarcoplasmic reticulum (SR) in two ventricular cardiomyocyte models (Ten Tusscher 2006 and Himeno HuVEC models). The authors show that SERCA and RyR play key roles in controlling SR calcium releases and that NCX plays an important role in generating DADs, with dual effects in the Ten Tusscher model. Finally, the authors demonstrate that sufficiently large areas with DADs can induce

ectopic/triggered activity in multicellular simulations.

The work is largely confirmatory, corroborating previous experimental and computational studies, but is methodologically sound, mostly clearly presented, and provides an interesting overview due to its parameter sensitivity analysis.

AR: We thank the reviewer for this assessment of our work.

The following aspects should be considered:

1. The initial analyses on the reduced calcium subsystem do not add much to the present manuscript. As indicated, the conditions required to generate SCRs are highly unphysiological, the method did not work in the HuVEC model and the importance of SERCA and RyR is obtained under much more relevant conditions during the subsequent analyses. Thus, the impact of this first section of the manuscript for the remainder of the story is negligible. The authors should consider removing the Section 2.1 and the corresponding methods section from the manuscript to improve focus and readability.

AR: We have modified it in the draft, and moved this section to the Supporting Information section.

2. The additional spontaneous calcium releases during the AP plateau observed in the TP06 model appear rather unphysiological to this reviewer. Is there any evidence that such releases occur at these depolarized potentials? While late phase-3 EADs have obviously been documented, these tend to occur at much more negative potentials.

AR: The additional "spontaneous calcium releases" during the AP plateau, often referred to as Late Ca2+ releases (LCRs), are different from the spontaneous Ca2+ releases (SCRs) leading to DADs. These LCRs are due to "late" triggered opening of RyRs by LCCs, during the plateau phase of AP. These LCRs have not been reported in most of the DAD studies, but they can coexist with DADs [see Ref. 56 (Shiferaw *et al.*) in the Manuscript]. In the TP06 model LCRs arise owing to the enhanced plateau phase LCC current and early recovery of the RyR from inactivation. These LCRs were reported in experiments; see, e.g., Refs. 55 and 57 of the manuscript (from Fowler *et al.*), which highlight the presence of late Ca2+ releases (LCRs) and ripples during the systolic phase of AP; and the observed LCRs occur because of random openings of LCCs during the plateau phase of the AP.

2b. The antiarrhythmic effects of an increase in NCX also appear to be independent of these LCRs. Indeed, since NCX primarily operates in forward mode during a normal heart beat, an increase in NCX would promote additional calcium extrusion. Thus, the sensitivity of RyR to (SR) calcium load (determined by overall calcium loading) and trigger dyadic calcium is likely a more important determinant of the effects of NCX upregulation. Indeed, previous work has shown a similar protective effect of NCX upregulation in a mouse ventricular cardiomyocyte model, independent of LCRs (Lyon et al. Front Physiol 2021).

AR: The enhanced NCX in forward mode could deplete the Ca load of the cytosol, which therefore could reduce the Ca load of SR and overall reduce the probability of DADs; however, the Late Ca2+ releases (LCRs) along with the forward mode NCX will aggravate the forward mode of NCX and therefore the depletion of the Ca storage inside the SR; see, e.g., Fig. 17 in the Supporting Information; a small LCR has pushed the NCX in the forward mode and therefore exaggerated the Ca removal from myocyte and reducing the overall Ca overload.

The enchancing of the NCX model did not lead to eliminiation of DADs in the HuVEC15 model but did eliminate DADs from the TP06 model; as these LCRs can have signifiant impact on the SR calcium store, we concluded that the LCRs could play a role in reducing the overall Ca overload and therefore the elimination of the DADs.

We have updated our conclusions accordingly (see lines 471-472) and cited the above-mentioned article by Lyon, et al..

3. Given that many studies have been conducted in this area and many of the aspects mentioned in the manuscript have been discussed previously, **a better comparison to previous literature is needed**. Example include, but are not limited to:
- The role of subcellular calcium releases (sparks / waves) and stochasticity in DAD generation and the limitations of deterministic common pool models are briefly mentioned in the limitations in generic terms, but key results from previous detailed models (e.g., work by Dr. Colman in PLoS Comput Biol, and studies by Dr. Sato) confirming the current results should be mentioned.

AR: As suggested we have added a few lines in Section, Discussion and Conclusion; see lines 484-485 and lines 491-493.

4) Line 355: Indeed, the most common perception is that increased NCX would promote DADs. However, the dual role on DAD amplitude and calcium load has previously been discussed in several studies (e.g., Heijman et al. J Cardiovasc Pharmacol 2015)

AR: To address this we have added a few lines [see line number (471-472)]; in our study we discuss the possible role of the LCRs to exaggerate the Ca unloading from SR, via NCX and inhibition of DADs from the myocyte.

5) The importance of source and sink interactions could be discussed in more detail. A key manuscript in this regard is already briefly cited (ref 53), but not discussed in any detail.

AR: The source and sink dependence is a separate study in itself, therefore, we have cited and Referred to the appropriate reference regarding that, in the Section “Discusion and Conclusions” (see line: 485-486).

Minor:

1. Please ensure that figures are presented and cited consecutively. At present the order at the end of the manuscript appears largely random. In the text, Figure 18 is cited after Figure 12 (line 368).

AR: The latter Figure was not significantly important to be kept within the main text; therefore, we have moved it to the Supplementary Information, and that is why these numbers are apart.

2. For figures 8 and 9, it would be helpful to have both in the same orientation. At present, one is in landscape and the other in portrait orientation, despite their identical structure.

AR: We have changed this in the new version of the mamuscript.

3. Line 257: “we infer that the TP06 model should be capable of triggering DADs and that the addition of the RyR significantly reduces” Change to “the addition of additional RyR-mediated calcium leak significantly reduces…”

AR: We have replaced this in the updated version of the manuscript.

******************************** **Reviewer #3:** **

Roshan and Pandit present a simulation-based investigation on DADs in human ventricular myocytes. Many simulations results are presented, by using two computational models, and some key factors in the origin of these proarrhythmic events are highlighted. In my view the scope of the study is not completely well defined. From the title it seems the focus would be on the comparison of two models. If so, when presenting and discussing the results it should be highlighted mainly strengths and weaknesses of the two models, which results proved to be consistent and can provide robust hypothesis about what happens in real cardiomyocyte and in case of non-concordant results between the models clarify which of the two is consistent with experiments or if this leaves open questions on the interpretation…

If, on the other hand, the aim is to add knowledge about DADs mechanisms under calcium overload and/or prevention, the open research questions could be better explicited, maybe starting from the title.
Here are a list of specific comments/observations.

AR: We thank the reviewer for this assessment of work.

Introduction:

• Line 9: human-cardiac-myocyte models: I would not talk of ‘models’ since, to my understanding, the reference is about experimental data and, in this paper, ‘model’ is always used to indicate computational/mathematical models.

AR: We have addressed this in the updated version of the Manuscript by referring to mathematical models.

• Line 32-34: use of acronymous TP06 and HuVEC15 without having introduced them (only in the abstract and after at lines 45-46)

AR: We have changed this in the updated version of the manuscript.

• Line 56: the choice of the models to be compared deserves some discussion. Is it possible to explain why only a few models are capable to reproduce DADs? How were the two selected among them? More recent, and likely more reliable, models of human ventricular AP have been published (e.g.: O'Hara-Rudy, Tomek-Rodriguez, Bartolucci-Passini-Severi). In particular, Bartolucci et al. claimed that their model '…allows reproducing delayed after-depolarizations (DADs), which could not be reproduced with the original ORd formulation'. It would have been interesting to compare this model too.

AR: Usually, a model with the capability of producing, Spontaneous Ca release, can produce DADs. To figure out the model to use, Ref. 37, by Fink *et al.,* proposed a method of reduced the Ca subsytem analysis, which we performed on several models, and found only few models, e.g, TP06 and HuVEC15 that can give DADs.

In the O'Hara Rudy model and Tomek-Rodriguez model the RyR dynamics is multiplicatively dependent on the I_CaL and, as I_CaL remains shut during diastole, these models cannot lead to spontaneous diastolic Ca releases and hence DADs (see Ref. 37).

Bartolucci-Passini-Severi model, which came in 2020, is similar to HuVEC15 model it can also produce DADs and EADs. We were not aware of this model, when we started conducting our study. To perform these test and simulations requires significant amount of analysis with isolated myocytes and tissue simulations, therefore, we will consider this in future studies.

• In my opinion the overview of principal results at the end of the introduction is not needed. It would be better to further detail/clarify the objectives.

AR: We have addressed this in the updated version of the manuscript.

• Line 77: definition of sections 2 and 3 is quite superimposed, maybe here 'presentation' instead of 'discussion'?

AR: We have addressed it in this updated version of the manuscript.

Methods

• A very short description of Markov model used in TP06 to model RyR would be useful

AR: The RyR model in the TP06 is deduced from the Markov model from Shannon et al. 2004 (see Ref. 38 in the Manuscript); and the deduced equation is Eq. (11) in the Supplementary Information. Therefore, we did not include the entire Markov model.

• Line 97: definition of CaRU is missing

AR: We have addressed this in the updated version of the manuscript.

• Line 104: please make it explicit that the model is an isotropic monodomain.

AR: We have addressed this in the updated version of the manuscript.

• Line 114-16: why different solvers for Vm and gating variables?

AR: Traditionally using forward Euler suffices for the time integration of the diffusion part of the PDE, for the cardiac tissue monodoamin equation (V_m); however, in the electrophysiology, the ODEs for the gating variables are linear and stiff ODEs, which require Rush-Larsen or Generalised Rush-Larsen schemes, which are more accurate and efficient methods (see Refs. 39 and 40 in the Manuscript).

• Line 119: HUVEC15 capital U here

AR: We have addressed this in the updated version of the manuscript.

• Line 125: 2cm of thickness seems much larger than any cardiac tissue

AR: The thickness of normal human ventricular wall varies between 1.2 cm; but in certain conditions such as hypertrophy the ventricular wall thickness can grow upto 2.0 cm or more; see the Ref. below.

```
@article{bois2017comparison,
  title={Comparison of maximal wall thickness in hypertrophic cardiomyopathy
differs between magnetic resonance imaging and transthoracic echocardiography},
  author={Bois, John P and Geske, Jeffrey B and Foley, Thomas A and Ommen, Steve
R and Pellikka, Patricia A},
  journal={The American journal of cardiology},
  volume={119},
  number={4},
  pages={643--650},
  year={2017},
  publisher={Elsevier}
}
```

• Why in figure 2 is the R4 case not reported? I suggest to add in the figure legend R1-R4 to better identify the 4 cases

AR: The cases R1-R4 in table 2, all are not presented in the figure 2; instead the cases R1-R4 in table 2, go in accordance with Figure 16 in the Supplementary Information, where we use G_CaL vs G_Kr data to create a linear fit to obtain the required G_kr for a given G_CaL value. This fit was later used in the phase diagrams for the TP06 and HuVEC15 models.

• Figure 2: the actual increase (overload) of calcium seems quite small in the HuVEC15 model. Does this mean that in this model DADs are not due to calcium overload?

AR: Due to the compartmentalization in the HuVEC15 model, the compartment responsible for spontaneous calcium release (SCR) from through RyR is Srrl; however, the corresponding compartment in the TP06 model is SR. Owing to different compartmentalization approaches, the absolute value of Ca in SR from TP06 is higher than SRrl from HuVEC15; but it does not mean that calcium overload is not neccessary for DADs to appear. To put it in another way, the sensitivity of RyR for Ca in SRrl in the HuVEC15 model is higher in comparison to that in the TP06 model.

• Line 165: which Appendix? Why only TP06?

AR: We present reduced Ca subsytem only for the TP06 model as we have used the continuation algorithms to analyse this model.

• Section 1.4: What is the value of the analysis of a calcium subsystem in which the main calcium current (ICaL) is neglected and I_NaCa artificially reversed by setting completely unphysiological sodium concentrations? It is not clear to me which results of this analysis have been used to inform the subsequent ones. If anything is really strictly required, I would withdraw this part.

AR: The SCRs are due to spontaneous Ca transients (releases) leading to DADs. If this condition for SCR persists in a reduced model, the sustained Ca transients can occur leading to Ca oscillations. Based on this idea, Ref.37, suggests that Ca oscillations in a reduced Ca subsystem from the model itself could be a good predictor of DADs in the full model.

From Ref. 37, “*The physiological rationale for investigating the subsystem was that the initial trigger of DADs was coming from within the cell, i.e., from within the Ca2+ subsystem, when the membrane potential was at the resting potential*”.

In the reduced models, the Ca overload is often induced by overloading Na_i, which reverses the mode of operation of NCX. Using numerical continuation, we obtained the required Ca concentrations in the cellular compartments around which Ca oscillations will start and therefore DADs will occur.

This way of the analysis was to test, the TP06 model’s capability to give rise to DADs, and also the changes required in the model to produce DADs.

For example, using the Fink *et al.* Hypothesis, we have shown that, in the TP06 model the required value of Ca in SR to produce DADs is very high (unphysiological); however, introducing the additional RyR leak, could reduce the Ca overload requirement significantly and in the physiological range (see Fig. 19 for a comparison in the updated version of the Manuscript).

Results

• Figure legends are missing! (at least in the pdf provided to reviewers)

AR: This happened because journal wanted the figures to be uploaded separately. In the updated version of the manuscript, we have fixed that.

• Line 219: Ca2+ subsystem (see Subsec. 2.1): but we are in Subsec 2.1 should it be Subsec 1.4?

AR: We have fixed it in the updated version of the manuscript.

• Section 3.1: see comment above. How can this analysis provide strong support for the occurrence DADs? DADs are, by definition, AFTERdepolarizations, occurring 'after' normal depolarizations. An autonomous system, with no external stimulation and no normal depolarizations is a completely different system.

AR: The SCRs are due to spontaneous Ca transients (releases) leading to DADs. If this condition for SCR persists in a reduced model, this will lead to a the sustained Ca transients and therefore Ca oscillations. Based on this idea, Ref.37, suggests that Ca oscillations in a reduced Ca subsystem from the model itself could be a good predictor of DADs in the full model.

From Ref. 37, "*The physiological rationale for investigating the subsystem was that the initial trigger of DADs was coming from within the cell, i.e., from within the Ca2+ subsystem, when the membrane potential was at the resting potential*".

• Section 3.1: this is a bit confusing. How were selected the parameters to be tuned? Which values were used to obtain different DADs types? Apparently this should go after the results of sensitivity analysis.

AR: In Section 3.1, we varied all the ion channel conductances along with other parameters such as V_maxup, and V_rel, in a range; in particular, we chose random scale factors, for the maximal conductances and the calcium fluxes, from a log-normal distribution that has a median value of 1; and we use the standard-deviation parameter σ = 0.1 to control the ranges of variation for these parameters. This techniques was borrowed from the Sec 2.4 (method for parameter sensitivity analysis). For clarity we have introduced this description in Section 3.1 in the updated manuscript [see line 217-224].

• Section 3.2: It seems to me that the most important 'output' with respect to which the sensitivity analysis should be performed is the occurrence of DADs, rather than their

frequency and amplitude (although amplitude is relevant too). A logistic regression approach would be more appropriate then (see other publications by the Sobie's Lab).

AR: This could be one of the approaches; however the phenomena seems more complicated. For example, for a given G_CaL factor (rate of Ca overload), if we change (increase or decrease) the parameter NCX by a factor, for reduction in NCX, we observe DADs of small amplitude but higher frequencies; however, if we increase NCX, we get an increase in the DAD amplitude, but with reduced frequency of the DADs. Therefore, describing them in terms of frequency and amplitude was important (in the mathematical models we considered).

• Line 292: in HuVCE15 Vrel is the most sensitive parameter for DAD frequency, but from fig.4 it seems to be G_CaL.

AR: The Ca overload is a necessary requirement for the DADs to be observed. The G_CaL in the sensitivity analysis shows up as the most crucial parameter; however, a change in the Ca overload method (e.g., Ca overload using Na_i overload) could eliminate the G_CaL dependence; therefore, G_CaL may not be a robust sensitive parameter. But the dependence on NCX, V_rel, and V_maxup is robust irrespective of the Ca overload method. To address this we have included it in the updated manuscript (see line numbers 257-261).

• Line 310-316: I cannot follow the description of this result, find where the 'biphasic' dependence on SKNaCa is shown, etc. Please double check!

AR: By biphasic we mean the following: Initally the effect of S_KNaCa is necessary for the occurrence of the DADs; an increase in S_KNaCa leads to an increase in the DAD amplitude, which continues upto a point. A further increase of S_KNaCa could make the DADs vanish completely (and have zero amplitude). Therefore, we call this renponse biphasic; it can be seen partially in the phase diagram. Therefore, we have improved it in the updated version of the manuscript.

• In figure 6(b) it is not clear what is reported into the x-axis, since above it is reported SKNaCa=2.

AR: We have changed this in the updated version of the manuscript.

• Line 405, description of figure 14 seems to not correspond to the figure itself

AR: We have fixed this in the updated version of the manuscript.

• Section 2.7: all the 1D/2D/3D simulations are carried out (if I understood correctly) assuming groups of identical cells, close each other, generating exactly the same kind of DADs at exactly the same time, when simulated as isolated cells. This obviously makes

synchronization and propagation of DADs much more likely than in real tissues where a huge heterogeneity is in place.

AR: In the common pool models, the SCRs are synchronized by the previous AP; once the focal excitations emerge the cell-to-cell variation does not play a major role in wave propagation and interactions.

Discussion

• Line 446: capital U

AR: We have fixed this in the updated version of the manuscript.

• Line 477: delete for

AR: We have introduced this correction in the updated version of the manuscript.

• Line 515: are figures shown for only the case of clumps of one DAD type?

AR: Yes in each simulation we have considered one DAD type at once.

• Line 532: what is Paper II?

AR: We had planned to submit two back-to-back related manuscripts, which we call paper i and paper ii, where, paper i, is the current manuscript, the other manuscript i.e. paper ii is the extension of this manuscript. Later, we decided two submit these two manuscripts independently, therefore mentioning paper ii was unnecessary here, so, we have removed it from the updated version of the manuscript.

• Line 540-546: please split the sentence to make it more readable

AR: We have fixed this in the updated version of the manuscript.

Supplementary Information

• Eq 5. In **Supplementary Information**: is it different from the original TP06 formulation?

AR: This set of equations is the same as in the original TP06 equation; these equations were put here, to complete the description of Section “The Ca 2+ subsystem and numerical continuation”.

• Line 711: delete Ref and “U” in HUVEC15

AR: We have fixed it in the revised mansucript.

- Description of video 3: Line 590-91 – is there an extra sentence?

AR: We have fixed it in the revised mansucript.

- Line 700: Vrel*0.000075ms^-1???

AR: The equations (Eq. 21 and Eq. 22) for the Ca release and RyR leak, from TP06 and HuVEC15 models, utilize different units, therefore, we have rearranged the Eq. 22 to make it comparable to Eq. 21. We have changed it in the updated version of the manuscript.
