# Supplementary material for: Abnormal calcium release and delayed afterdepolarizations: A comparison of two mathematical models for human ventricular myocytes
Source: PLoS One. 2026 Feb 11;21(2):e0338873. doi: 10.1371/journal.pone.0338873 (PMC12893660; doi:10.1371/journal.pone.0338873)
Supplement: Attachment — Submitted filename: Authors_Respose_Review2.pdf [file pone.0338873.s014.pdf]

Dear Editors,

Thank you for your message below, for your invitation to revise our manuscript in the light of the comments of the Reviewers #1 and #2, and for their reports on our paper. We would like to thank them for taking the time to prepare their reports on our paper.

We are happy to see that Reviewer #2 has said that “All comments have been addressed.”

Reviewer #1 has listed some additional comments. We have now revised our paper in the light of these comments and have addressed *all the points raised* by the Reviewer #1 (see below) *again.* We hope, therefore, that our revised manuscript will now be accepted for publication in PLoS.

Thanking you and with our best wishes,

Yours sincerely,

Navneet Roshan [l'institut du thorax, Nantes Université, France]

and

Rahul Pandit [Department of Physics, Indian Institute of Science, Bangalore, India]

Response to Reviewers

The points raised by the Reviewers are shown in black. Our responses are given in blue; and the red color new text that we have added to the revised manuscript, to address the comments of the Referee, is shown in red.

Reviewer's Responses to Questions

Black color: reviewer comments

Blue : Author response

RED color: changes in the manuscript.

Comments to the Author

1. If the authors have adequately addressed your comments raised in a previous round of review and you feel that this manuscript is now acceptable for publication, you may indicate that here to bypass the “Comments to the Author” section, enter your conflict of interest statement in the “Confidential to Editor” section, and submit your "Accept" recommendation.

Reviewer #1: (No Response)

Reviewer #2: All comments have been addressed

---

2. Is the manuscript technically sound, and do the data support the conclusions?

The manuscript must describe a technically sound piece of scientific research with data that supports the conclusions. Experiments must have been conducted rigorously, with appropriate controls, replication, and sample sizes. The conclusions must be drawn appropriately based on the data presented.

Reviewer #1: Yes

Reviewer #2: Yes

---

3. Has the statistical analysis been performed appropriately and rigorously?

Reviewer #1: N/A

Reviewer #2: N/A

---

4. Have the authors made all data underlying the findings in their manuscript fully available?

The PLOS Data policy requires authors to make all data underlying the findings described in their manuscript fully available without restriction, with rare exception (please refer to the Data Availability Statement in the manuscript PDF file). The data should be provided as part of the manuscript or its supporting information, or deposited to a public repository. For example, in addition to summary statistics, the data points behind means, medians and variance measures should be available. If there are restrictions on publicly sharing data—e.g. participant privacy or use of data from a third party—those must be specified.

Reviewer #1: Yes

Reviewer #2: Yes

5. Is the manuscript presented in an intelligible fashion and written in standard English?

PLOS ONE does not copy edit accepted manuscripts, so the language in submitted articles must be clear, correct, and unambiguous. Any typographical or grammatical errors should be corrected at revision, so please note any specific errors here.

Reviewer #1: Yes

Reviewer #2: Yes

---

6. Review Comments to the Author

Please use the space provided to explain your answers to the questions above. You may also include additional comments for the author, including concerns about dual publication, research ethics, or publication ethics. (Please upload your review as an attachment if it exceeds 20,000 characters)

Reviewer #1: Abnormal Calcium Release and Afterdepolarizations: A Comparison of Two Mathematical Models for Human Ventricular Myocytes

This study uses computational simulations to investigate the electrophysiological conditions leading to delayed afterdepolarizations (DADs) in the human left ventricle. This effect is demonstrated using two different ODE-based models derived from published models by Ten Tusscher (TP06) and Himeno (HuVEC15). The authors detect changes in the DAD activity predicted by these models (represented using “phase diagrams”) as model parameters are altered. They then interpret these observations in terms of the mechanisms underlying DAD formation, which are associated with intracellular calcium dynamics. The implications of these behaviours is explored in multi-dimensional finite-element simulations.

OVERALL OBSERVATION

The subject matter of the paper is certainly interesting and I agree that a mechanistic understanding of DADs is useful, e.g., for the development of pharmacological treatments. The topic lends itself well to computational simulation, to resolve the roles played by the different system components and to explore hypothetical scenarios, as was done in this study.

We thank the Reviewer for this positive assessment of our work.

However, I feel that the study, as presented here, has significant shortcomings that prevent its conclusions from being truly convincing. I therefore cannot recommend this manuscript for

publication without major improvements.

In summary, my objections are along the following general themes:
1. Lack of a clearly formulated hypothesis to test. It is established from the outset that DADs arise from calcium dynamics. The aim of the paper is hence defined as “to delineate the role of the parameters on the DAD features [using two ventricular models]”, but I find this somewhat vague. The paper thus reads like “we’ll test several models and see if anything interesting comes out”. This lack of clarity of purpose transpires in the Discussion, where conclusions are drawn with regard to one model (the amended TP06), but not the other. Because the outcomes of the different models differ significantly, it is difficult to draw a convincing overall conclusion and to make a critical comparison of the models in the perspective of the real system. A clearer, more focused mission statement would help justify the choice of model(s) and tailor the analysis methodology more systematically.

2. questionable methodology and analysis. The main conclusions of the study rest on the interpretation of the “phase diagrams”, which arguably segregate the parameter spaces into qualitatively distinct regimes. As explained specifically below, I have reservations with regards to both (i) the definitions of the different phases and (ii) the analysis and conclusions drawn from the (arbitrarily chosen?) set of sections through the phase space. Although I appreciate the difficulty of rigorously defining regimes of behaviour, these challenges must be addressed critically for the results to be instructive.

3. overambitious scope. Although understanding DADs at the cell level must ultimately benefit understanding at the tissue and organ levels, combining all these levels within this study seems to me to be premature, especially as so many questions remain to be addressed at the basic level. To avoid confusion, I would encourage the authors to proceed more methodically before attempting to tackle the multi-scale problem.

AR: We thank the reviewer for bringing this out,

Many of the recent works (see Refs. (a - e) mentioned below) have shown that during Ca overload late and spontaneous calcium releases (LCR and SCR) both can be observed, which is against the idea that “ refractory period for Ca spark initiation after first Ca-induced Ca-release (CICR)”. These LCRs have been observed even in healthy cardiac myocytes. We hypothesize that these Ca overload induced LCRs and SCRs in a structurally intact heart, could dynamically present heterogeneous APD distribution that can act as a substrate for reentry and fibrillation.

With two model, we systematically explore how various

physiological factors including calcium load, SR leak, NCX activity, and compartmental diffusion affect SCRs and LCRs and the resulting membrane depolarizations. By comparing these two models under equivalent stimulation protocols and perturbations, we aim to identify the key determinants of DAD initiation, amplitude, and their ability to propagate and trigger PVCs in tissue-level simulations. This comparison provides insight into how model structure and complexity influence the emergence and evolution of arrhythmogenic events and helps delineate the minimal requirements necessary to reproduce various types of DAD behaviors.

To address this issue, we have modified our Manuscript including the Introduction and Methods to Results and Conclusion sections.

(a) Arrhythmogenic late Ca2+ sparks in failing heart cells and their control by action potential configuration, E.D. Fowler, et al., January 22, 2020, PNAS, 117 (5) 2687-2692 https://doi.org/10.1073/pnas.1918649117;

(b) Fowler ED, Kong CHT, Hancox JC, Cannell MB. Late Ca2+ Sparks and Ripples During the Systolic Ca2+Transient in Heart Muscle Cells. Circ Res. 2018 Feb 2;122(3):473-478. doi: 10.1161/CIRCRESAHA.117.312257. Epub 2017 Dec 27. PMID: 29282211; PMCID: PMC5796647;

(c) Atrial Remodeling and Atrial Fibrillation: Mechanisms and Implications, Stanley Nattel, MD, Brett Burstein, BSc, and Dobromir Dobrev, MDCirculation: Arrhythmia and Electrophysiology Volume 1, Number 1 (2008) https://doi.org/10.1161/CIRCEP.107.75456;

(d) NCX-Mediated Subcellular Ca2+ Dynamics Underlying Early Afterdepolarizations in LQT2 Cardiomyocytes Mingwang Zhong, Colin M. Rees, Dmitry Terentyev,· Bum-Rak Choi, Gideon Koren, Alain Karma, Biophysical Journal, Volume 115, Issue 6p1019-1032September 18, 2018.

(e) Alvarez JAE, Jafri MS, Ullah A. Local Control Model of a Human Ventricular Myocyte: An Exploration of Frequency-Dependent Changes and Calcium Sparks. Biomolecules. 2023 Aug 17;13(8):1259. doi: 10.3390/biom13081259. PMID: 37627324; PMCID: PMC10452762.

Model choice and constitution

RQ: The exact rationale for choosing TP06 and HuVEC15, as opposed to the other candidates, is not clearly and explicitly explained. The "Ca2+-oscillation hypotheses" are cited but without an explanation of the underlying principle. Despite similarities, the two chosen models differ markedly in terms of their structure and predictions. The reader may then wonder whether several models were used in the hope that one would yield appealing conclusions.

AR: To address this we have added following in the introduction section from line number 29-41

"Both TP06 and HuVEC15 are capable of exhibiting DAD-like behavior under appropriate conditions, which makes them suitable for exploring the parameter regimes and cellular conditions that promote or suppress arrhythmogenic DAD activities.

The HuVEC15 model has more refined representation of L-type calcium channels (LCCs) and RyRs, which more accurately captures the localized control of calcium-induced calcium release (CICR). Additionally, it incorporates a more elaborate calcium compartmentalization, separating the cytosolic space into multiple subdomains, such as the junctional space (jnc), intermediate zone (iz), and bulk cytosol (blk); moreover, the sarcoplasmic reticulum (SR) is also more compartmentalized and the ion channels such as the Na+/Ca2+ exchangers (NCX) and the LCCs are distributed across these compartments, enabling more compartmentalized electrogenic effects and intracellular calcium handling [24]"

RQ: The ability of TP06 to produce DADs relies on an alteration of the published model but the nature of that change is not explained or supported. The reader is directed to the Supplement, which I don't feel is appropriate, given the centrality of this issue to the paper. Without a proper clarification, justification, and validation of the alterations, it is difficult to establish that TP06 is a suitable platform for the present purpose. However, we learn only in the Discussion section that the TP06 amendment involves introducing a SR leak current. It is potentially misleading to even refer to the altered model as "TP06". Furthermore, as shown in Fig. 3, the DADs in the TP06 case coincide with the appearance of additional oscillations within the AP that, in the absence of explicit commentary, raises questions on the reliability of the model. Was this model included in the study simply because its apparent demonstration of the "protective" effect of NCX is appealing?

AR: To address this we have added following in the Methods section from line number 141-156

" The opening of the RyRs in the TP06 is modeled via the Ca-induced-Ca release (CICR). The calcium availability inside the SR stores (CaSR) and the subspaces outside the RyR, also called triggers (CaSS) both modulate the opening probability O of the RyR. If the trigger is not enough during the diastole, then Ca2+ sparks and DADs do not occur. Therefore, a small calcium leak, through the closed RyRs (see Refs. [36, 37]), suffices for [Ca2+]SS to reach the trigger threshold levels required for SCRs and DADs. Therefore, we add a small calcium leak (background SR Ca2+ release that is independent of the RyR opening probability) through the RyR channel, in the TP06 model, as follows:"

The appearance of additional oscillations during the TP06, AP 305 to 308

Note that the Vm oscillations during the plateau phase of the AP, in the TP06 305
model; these oscillations are present due to late Ca release, causing membrane 306
depolarizations via NCX. No such oscillations were present in HuVEC15 model

RQ: In the case of HuVEC15, the NCX distribution was adjusted, "to increase the amplitude of the DADs" (line 162). What were the targets? Is this recalibration of the original model justified? Can we be certain that the "phase diagrams" do not depend critically on the altered model calibrations?

AR: To re-emphasize the motivation for the choice of mathematical models, we have modified the Methods section (line: 146, 156)

Without any parameter modifications, the HuVEC15 model exhibits delayed 146
afterdepolarizations (DADs); however, their amplitudes remain subthreshold. In the 147
original model configuration, 90% of NCX ion channels are located in the bulk cytosol 148
(blk), with the remaining 10% distributed within the intermediate zone (iz), adjacent to 149
the RyRs (see Fig.1 in the main text). Notably, experimental and computational studies 150
suggest that up to 45% of NCX channels may be localized near RyRs [24]. To reflect 151
this, we redistributed the NCX channels in the model—allocating 25% near the RyRs 152
and 75% elsewhere. This adjustment enabled the model to reproduce experimentally 153
observed supra and subthreshold DADs. In the HuVEC15 model, the parameter that 154
controls this distribution is fNCX. In this study, we vary fNCX to investigate its 155
potential role in the origin of DADs

Chu L, Greenstein JL, Winslow RL. Modeling Na^{+}-Ca^{2+} exchange in the heart: Allosteric activation, spatial localization, sparks and excitation-contraction coupling. J Mol Cell Cardiol. 2016 Oct;99:174-187. doi: 10.1016/j.yjmcc.2016.06.068. Epub 2016 Jul 2. PMID: 27377851; PMCID: PMC5534334.

The Fink, *et al.,* 2008 model mentioned in the main text of the manuscript, borrowed most of the ion channel formulations from the TP06 model; therefore, it should produce qualitatively similar results to the TP06 model. The model of Iyer *et al.* 2008~\cite{}, model is capable of leading to SCRs and DADs; but it has not been used widely.

We choose to work with two specific models, TP06 and HuVEC15, because we can reproduce the range of the behaviors that we have highlighted above, in these models.

We have removed these model citations from the introduction for clarity.

• The ability of the TP06 model, to produce DADs relies on an alteration of the published model but the nature of that change is not explained or supported. The reader is directed to the Supplement, which I don't feel is appropriate, given the centrality of this issue to the paper. Without a proper clarification, justification, and validation of the alterations, it is difficult to establish that TP06 is a suitable platform for the present purpose. However, we learn only in the Discussion section that the TP06 amendment involves introducing a SR leak current. It is potentially misleading to even refer to the altered model as "TP06".

AR: The published TP06 cell model was developed to reproduce the healthy cardiac myocyte AP; however, to express the behavior of the cardiac myocyte during various pathological conditions we need to adapt the model; because the change is minimal, we still call the model the TP06 model. [We have included these equations along with the modifications, in the Methods Section.] The full fledged discussion of the Ca oscillation hypothesis can be found in Ref. Fink *et al.* 2011, because that study does not discuss the TP06 model, we performed the study using the same methodology to justify the need for the modifications in the model; however, that would divert the primary aim of our current work.

We have included in the methods section the amended Equations from the TP06 (page 141-156; Eq 5)

RQ: Furthermore, as shown in Fig. 3, the DADs in the TP06 case coincide with the appearance of additional oscillations within the AP that, in the absence of explicit commentary, raises questions on the reliability of the model. Was this model included in the study simply because its apparent demonstration of the "protective" effect of NCX is appealing?

AR: These oscillations in the membrane potential were present because of the Late Ca releases (LCRs), which depolarises the transmembrane potential via the forward mode of the NCX; these LCRs are handled qualitatively in the current model. we have added the following text to our revised manuscript (on line 239-241):

We also highlight the occurrence of membrane potential oscillations during the plateau phase of the action potential, these oscillations are are caused by late Ca releases (LCRs) see Fig. 16 in Supporting Information and Refs. [43, 44].

RQ: In the case of HuVEC15, the NCX distribution was adjusted, “to increase the amplitude of the DADs” (line 162). What were the targets? Is this recalibration of the original model justified? Can we be certain that the “phase diagrams” do not depend critically on the altered model calibrations?

AR: To address this point, we have added the following text to our revised manuscript (on page 6 line 164-169):

The HuVEC15 model has 90% of ion channels in the bulk cytosol region; and the remaining 10% are in the intermediate zone, (iz near RyRs see Fig1 in main text). However, the fraction of NCX ion channels localised near the RyRs could be up to 45% (see Ref. Chu *et al.* below). We redistribute these NCX, 25% near RyRs and 75% elsewhere. Without this change we cannot replicate experimentally observed suprathreshold DADs. The phase diagrams were obtained after accounting for these changes.

Chu L, Greenstein JL, Winslow RL. Modeling Na^{+}-Ca^{2+} exchange in the heart: Allosteric activation, spatial localization, sparks and excitation-contraction coupling. J Mol Cell Cardiol. 2016 Oct;99:174-187. doi: 10.1016/j.yjmcc.2016.06.068. Epub 2016 Jul 2. PMID: 27377851; PMCID: PMC5534334.

Analysis and interpretations.

RQ The two models TP06 and HuVEC15 are described in parallel rather than in sequence (Sec 2.1). Details of numerical integration are also given in parallel (Sec 2.2). I think it would help the logical flow if they weren’t so merged. No explanation is given as to why the analysis protocols

differ between the two models. For example, TP06 is solved using the Rush-Larsen scheme and HUVEC15 using the generalized Rush-Larsen scheme (line 108). What motivated these choices? Can we be certain that the differences in outcomes arise from intrinsic biophysical differences between the models rather than from details in their implementation?

AR: We have changed the description of the models, we have added the following text to our revised manuscript (on page 4, line no 74-88):

Except for a few minor differences, both models share most ion channels; however, their Ca2+ handling differs markedly. In the TP06 model, the sarcoplasmic reticulum (SR) is represented as a single compartment, and the intracellular space is divided into the subspace (SS) and the cytosol (CYTO).....

To address the difference in integration schemes, we have added the following text to our revised manuscript (on page 4, line no 93-96):

The formulation of ion channel dynamics differs between the TP06 and HuVEC15 models. The gating variables described using the Hodgkin–Huxley formalism were integrated with the Rush–Larsen method, whereas the ODEs of other forms are integrated using the generalized Rush–Larsen scheme (see Refs. [29,30] for comparison).

We have addressed the impact of modeling choices for example compartmentalizations, that has been, included in the **Introduction**, **Methods**, **Results** and **Conclusions**

RQ: Ca2+ overload is implemented by varying GCaL while also varying GKr to keep the APD constant. Was the impact of other model parameters on the APD (and indeed on the AP morphology) considered when choosing GKr as the correction factor? The AP morphology in Fig. 2 changes noticeably with GCaL, despite the GKr correction. Might the subsequent conclusions be affected by the precise definition of APD adopted?

AR: To address this point, we have added the following text to our revised manuscript (on page 12 line no 454-460):

Increasing GCaL increases the APD; to counterbalance this we increase GKr. For the initial few beats this balance is maintained; however, as the Ca overload builds up, after a few pacings, the myocyte fires LCRs, which then cause an extra increase in the APD via NCX. This increase

in APD is not unique to our study; this has also been found, e.g., in Ref. Fowler *et al. 2020* (below) shows an elongated AP because of similar LCRs. The APD we talk about holds true for the first few beats, before the LCRs occur and cause APD elongation.

E.D. Fowler, N. Wang, M. Hezzell, G. Chanoit, J.C. Hancox, & M.B. Cannell, Arrhythmogenic late Ca2+ sparks in failing heart cells and their control by action potential configuration, Proc. Natl. Acad. Sci. U.S.A. 117 (5) 2687-2692, https://doi.org/10.1073/pnas.1918649117 (2020).

RQ Given the centrality of the “frequency” and “amplitude” values in the generation of the phase diagrams, an appraisal of these quantities in terms of real-life DADs would be useful, if only to help justify the chosen model alterations.

AR: To address this point, we have added the following text to our revised manuscript (on page 7, line 238):

The frequency here indicates the recovery and re-firing of the RyRs, from refractoriness. It has been suggested that this frequency depends on the replenishing rate of the SR Ca (see Ref. Fowler *et al.* below).

E.D. Fowler, N. Wang, M. Hezzell, G. Chanoit, J.C. Hancox, & M.B. Cannell, Arrhythmogenic late Ca2+ sparks in failing heart cells and their control by action potential configuration, Proc. Natl. Acad. Sci. U.S.A. 117 (5) 2687-2692, https://doi.org/10.1073/pnas.1918649117 (2020).

RQ: More specifically, Fig. 3 shows that both the time period and amplitudes of consecutive DADs are generally not constant. How does this compare to the real-life case? Are the amplitude and frequency values, “averaged” over (presumably?) the steady-state even meaningful characterisations?

AR: Indeed, real- life DADs are averaged outcomes of the stochastic behaviors of thousands of RyRs. However, averaged or deterministic behaviour is often useful in identifying crucial physiological factors involved in the arrhythmogenesis.

RQ: I do not think that defining the DAD frequency equal to zero in the supra-threshold regime is meaningful; it can even be misleading. Likewise, what is the meaning of “frequency” in the case where only one (or even two!) DAD occurs? The concept of frequency really only makes sense in the context of regular cycling, but it loses clarity when the number of full cycles is very small (at most two in the present case). The “multi-blip” DADs are presented as a class distinct from the single-blip DADs. Is this qualitative distinction objectively justified, or could they simply result from the time difference between consecutive blips simply exceeding the pacing period? These uncertainties can generate skepticism on the interpretations of the “phase transitions” in Figs 6 and 7.
Is the casting of these transitions as “Hopf bifurcations”, mentioned in the Discussion section for the first time, properly supported?

AR: We have not considered the suprathreshold DADs in frequency plots and the sensitivity analysis, as the occurrence of suprathreshold DADs could change the overall Ca loading rate by modifying the apparent stimulation frequency of the myocytes. To calculate the frequency, we count the number of times DADs appear in the last 10 pacings and average this frequency over 10 cycles.

The multi-blip DADs occur during very-high Ca overload and high SERCa pump dominance over NCX. However, subthreshold DADs are expected when the Ca overload is lower side . For a discussion of the Hopf bifurcations, see the Supplemental Material.

RQ: Sensitivity analysis results Sec 3.2. Line 240 reads “for the HuVEC15 model, Vrel is the most sensitive parameter for the DAD frequency”. This doesn’t seem right, as the GCaL sensitivity is approximately twice as large, and KNaCa is negative and of similar magnitude.

AR: To address this point, we have added the following text to our revised manuscript (LINE NUMBER 261-283):

Note that in Figs.4 (a) and (c), Ca2+ overload is a necessary condition for DADs to occur. The appearance of GCaL as a sensitivity factor suggests that the rate of Ca2+ overload is a key determinant of DAD frequency. However, when the method of Ca2+ overload changes, e.g., when it is induced by Nai accumulation the dependence on GCaL could disappear from the sensitivity analysis. Therefore, GCaL may not be a robust sensitivity parameter across different Ca2+ overload methods.

RQ: Is the casting of these transitions as “Hopf bifurcations”, mentioned in the Discussion section for the first time, properly supported

AR: Indeed this we find not properly supported in the main tex. The hopf bifurcations theory is mentioned in the supplementary section in detail therefore we have removed it from the Conclusion section.

• The conclusion of results Sec 3.3 is the “important difference” between the TP06 and HuVEC15 results with regard to the reentry of the “normal AP” phase at large SKNaCa. However, I do not find this conclusion very convincing. Although Figs 6 and 7 are described as “representative sections” of the parameter space, there is no indication that they provide an objective overall understanding of the entire space. How “representative” are these sections? It is stated that there is such a reentry for TP06 but not HuVEC15, but such reentry is not apparent in Fig. 6d. Can we have confidence in these statements, based on the very coarsely pixelated phase diagrams of Figs. 6 and 7? If the occurrence of a transition is apparently so dependent on the trajectory in the parameter space, can we be certain that the TP06 and

HuVEC15 differ fundamentally (line 314: “this protective mechanism is only present in the TP06 model”)?

We have broken this comment into three parts, which we address separately below.

RQ: The conclusion of results Sec 3.3 is the “important difference” between the TP06 and HuVEC15 results with regard to the reentry of the “normal AP” phase at large SKNaCa. However, I do not find this conclusion very convincing. Although Figs 6 and 7 are described as “representative sections” of the parameter space, there is no indication that they provide an objective overall understanding of the entire space. How “representative” are these sections?

AR: We have used the term “representative” because four sensitive parameters means, we should present a four dimensional phase diagram, but in each phase diagram we show two parameters at once and keep two other parameters fixed. To address this issue, we have added the following line in the 292-296,

Since there are four sensitive parameters, the full phase diagram is four-dimensional and cannot be fully represented on a 2D page. Therefore, we vary two parameters at a time while keeping the other two fixed, selecting combinations that best illustrate the phase diagram. These phase diagrams reveal the trends in DAD patterns across the parameter spaces of the TP06 and HuVEC15 models, as shown in Figs.6 (a)–(e) and Figs.7 (a)–(e), respectively. Note that, in this subsection, the average DAD amplitude was measured for the final 10 pacing cycles.

RQ: It is stated that there is such a reentry for TP06 but not HuVEC15, but such reentry is not apparent in Fig. 6d.

AR: To address this point, we have added the following text to our revised manuscript (on page 9, 307 to 309):

This reentry exists only up to the moderate levels of S_GCaL (<= 2.5); indeed, for a very high level of S_GCaL (> 2.5), the phase diagram does not show the return of the suprathreshold DADs to a no-DAD regime, when we increase S_KNaCa.

RQ: Can we have confidence in these statements, based on the very coarsely pixelated phase diagrams of Figs. 6 and 7?

AR: The phase diagram is pixelated because the complexity and nonlinearity of the mathematical models that are used does not allow for a very fine exploration of parameter space given current computational resources. Such phase diagrams are often seen in highly nonlinear and complex systems (see, e.g., Ref. Song *et al.*]).

[Song Z, Ko CY, Nivala M, Weiss JN, Qu Z. Calcium-voltage coupling in the genesis of early and delayed afterdepolarizations in cardiac myocytes. Biophys J. 2015 Apr 21;108(8):1908-21. doi: 10.1016/j.bpj.2015.03.011. PMID: 25902431; PMCID: PMC4407256.]

RQ: If the occurrence of a transition is apparently so dependent on the trajectory in the parameter space, can we be certain that the TP06 and HuVEC15 differ fundamentally (line 314: “this protective mechanism is only present in the TP06 model”)?

AR: Yes indeed the TP06 and the HuVEC15 models differ significantly in terms of the approach they have taken to model the intracellular Ca dynamics. To address this point, we have added the following text to our revised manuscript (on introduction page line 74-88):

Except for a few minor differences, both models share most ion channels; however, their Ca2+ handling differs markedly. In the TP06 model, the sarcoplasmic reticulum (SR) is represented as a single compartment, and the intracellular space is divided into the subspace (SS) and the cytosol (CYTO). By contrast, the HuVEC15 model partitions the SR into two regions: the uptake compartment, SRup, and the release compartment, SRrl. These are coupled by diffusion flux from SRup to SRrl. Moreover, HuVEC15 features more detailed intracellular compartmentalization: the cytosol is subdivided into junctional (jnc), intermediate zone (iz), and bulk cytosol (blk) compartments. This level of detail permits modeling of heterogeneous ion-channel distribution across

Chu L, Greenstein JL, Winslow RL. Modeling Na^{+}-Ca^{2+} exchange in the heart: Allosteric activation, spatial localization, sparks and excitation-contraction coupling. J Mol Cell Cardiol. 2016 Oct;99:174-187. doi: 10.1016/j.yjmcc.2016.06.068. Epub 2016 Jul 2. PMID: 27377851; PMCID: PMC5534334.

Although we observe slightly different behavior from different model, but all these behaviors have been observed across the existing literature.

Reviewer #2: I thank the authors for addressing my comments in this revised version. I do not have any additional suggestions.

---

7. PLOS authors have the option to publish the peer review history of their article (what does this mean?). If published, this will include your full peer review and any attached files.

If you choose “no”, your identity will remain anonymous but your review may still be made public.

Do you want your identity to be public for this peer review? For information about this choice, including consent withdrawal, please see our Privacy Policy.

Reviewer #1: No

Reviewer #2: No
